# The International Medical Annual

**Published:** 1901-05

**Authors:** 


The International Medical Annual. A year book of treatment and practitioners' index. Thirty-four contributors, American and foreign. Nineteenth year. 1901. Octavo, 682 pages. New York and Chicago: E. B. Treat & Co. 1901. [Price, $3 00.]

The nineteenth annual issue of this popular work is before the profession for its approval and acceptance, and is a worthy successor of a long line of valuable medical contributions. The present volume keeps abreast of the times and some changes are noticed looking toward that end; thus in the department of therapeutics an excellent article is added on toxins and antitoxins, the conjoint work of Professor McFarland, of Philadelphia, and Dr. William Murrell. Another very important chapter is that on the influence of light on the various tissues of the body, also by Dr. Murrell. *X*-ray work in medicine and surgery is ably handled by Dr. MacIntyre, of Glasgow; Color-blindness, by Dr. Edridge Green, and another special article by Mr. Turner, on Dental and oral surgery. The other departments are up to the usual standard; that on tuberculosis having been undertaken by Professor Puata of the University of Perugia, Italy.

The plates, fourteen in number, have been handsomely executed in colors and add materially to the value of the annual; forty-five wood engravings are interspersed throughout the volume, elucidating the text and simplifying the description of various operative procedures. In appearance the volume is similar to Treat's well-known classics. W. C. K.

